# Tunable long persistent luminescence in the second near-infrared window via crystal field control

**DOI:** 10.1038/s41598-017-12591-1

**Published:** 2017-09-29

**Authors:** Jianmin Nie, Yang Li, Shanshan Liu, Qiuqun Chen, Qi Xu, Jianrong Qiu

**Affiliations:** 1 State Key Laboratory of Luminescent Materials and Devices, School of Materials Science and Technology, South China University of Technology, Guangzhou, 510640 China; 2 Guangdong Provincial Key Laboratory of Fiber Laser Materials and Applied Techniques, South China University of Technology, Guangzhou, 510640 China; 3 School of Physics and Optoelectronic Engineering, Guangdong University of Technology, Guangzhou, 510006 China; 4 State Key Laboratory of Modern Optical Instrumentation, College of Optical Science and Engineering, Zhejiang University, Hangzhou, Zhejiang, 310027 China

## Abstract

Construction of an active composite as a biomarker with deeper tissue penetration and higher signal-to-noise ratio (SNR) is of great importance for the application in bioimaging. Here, we report a strategy for tuning the emission bandwidth and intensity via crystal field control in long persistent phosphors (LPPs). Ni^2+^-doped Zn_1+y_Sn_y_Ga_2−x−2y_O_4_ phosphors, with a tunable emission band peaking from 1270 to 1430 nm in the second near-infrared (NIR) window, have been successfully prepared. Such featured materials have the advantages of low absorption and scattering as well as more efficient tissue penetration. The emission spectra can be controlled by tailoring the local crystal field around the activator precisely via substitution of Zn and Sn for Ga. Moreover, with high resolution and weak light disturbance, these developed multi-band afterglow phosphors exhibit great application potential in advanced optical imaging.

## Introduction

In the recent years, biomarkers have become a hot topic and drawn much attention in the application of *in vivo* bioimaging^1,2^, since high precision and resolution for imaging technology are in great demand. Correspondingly, due to superior performance of long persistent phosphors (LPPs), they are recognized as the suitable materials to satisfy the requirement, i.e. the exclusion of external illumination which removes the possibility of autofluorescence from background noise^3,4^. Just like other luminescent biomarkers, the applications of LPPs are also strongly dependent on various parameters, including emission bandwidth and intensity. Higher intense afterglow emission and lower transmission loss are essential for improving the signal-to-noise ratio (SNR) in the process of optical signal acquisition in *in vivo* imaging^5,6^. Indeed, near-infrared (NIR) light can penetrate biological tissues, such as skin and blood, more efficiently than visible light, while the light in the second NIR window (1000-1400 nm) has lower absorption, lower scattering and more efficient tissue penetration than the light in the first NIR window (650-950 nm)^7,8^. Thus, optical materials with tunable spectra are still highly desirable; especially long persistent phosphors with NIR emission. Unfortunately, an instructive methodology of purposefully tuning the afterglow properties is still missing^9–12^.

Perfect crystals did not exist in reality. Foreign atoms or imperfections make materials highly interesting in the aspect of functional and structural systems. Therefore, these foreign atoms are either chosen as the optical and electrical activators to offer the anticipative application, or as the purposely introduced dopant to vary the local site symmetry^13,14^. Hence, we suggest a strategy of element substitution to tune the operational waveband and emission intensity. This route potentially achieves the control of crystal field surrounding the activators and increases the SNR during the process of optical signal acquisition. Experimentally, we demonstrate that the long persistent phosphorescence in Zn_1+y_Sn_y_Ga_2−x−2y_O_4_: xNi^2+^ with the tunable emission band peaking from 1270 to 1430 nm is tailored by controlling local crystal field around the activator, which allows extensive control of emission band in the second NIR window.

## Results and Discussion

### Long persistent phosphorescence in the second near-infrared window

**Figure 1:**
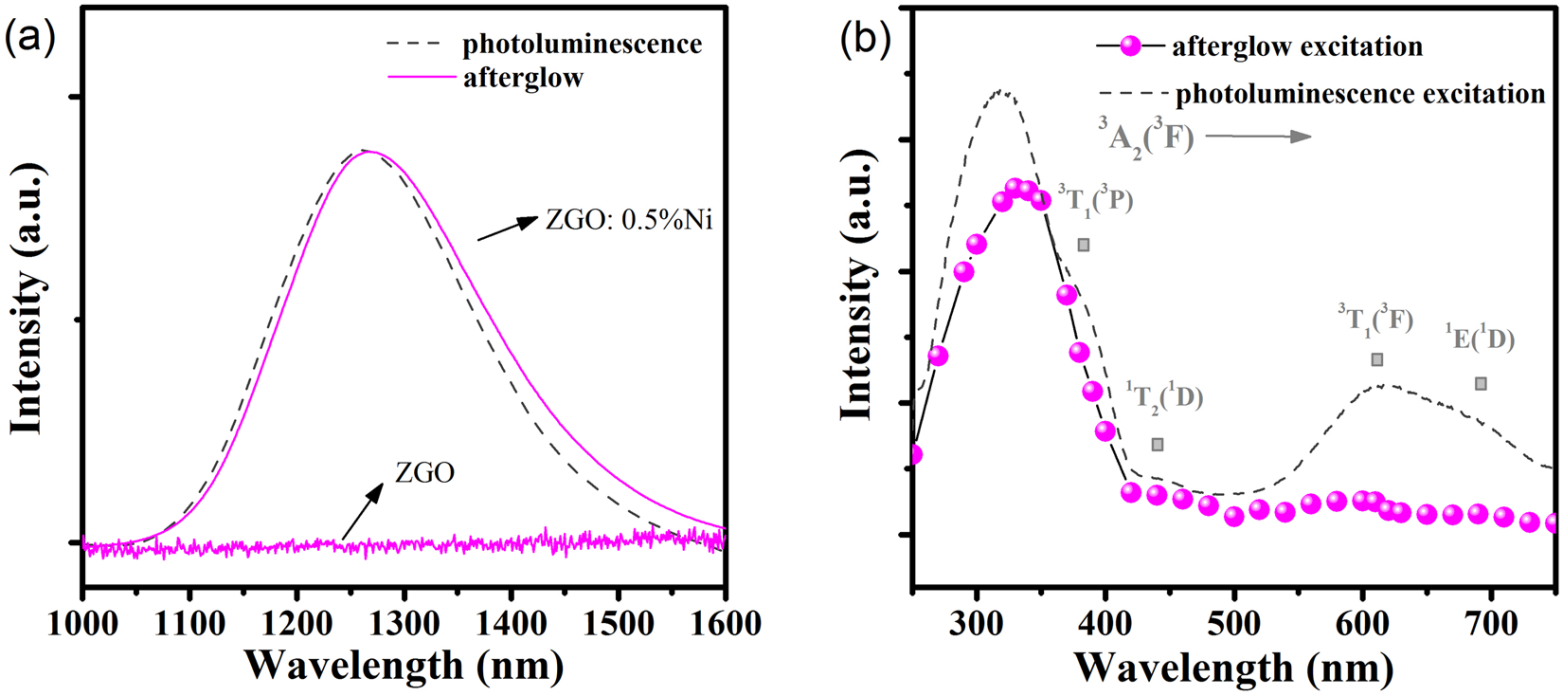
(**a**) Afterglow spectra of Ni^2+^-doped (ZGO: 0.5%Ni) and non-doped (ZGO) ZnGa_2_O_4_ phosphors after the irradiation by an ultraviolet lamp for 10 minutes, and photoluminescence spectrum of the ZGO: 0.5%Ni phosphor under excitation at 320 nm. (**b**) Photoluminescence excitation spectrum and afterglow excitation spectrum of ZGO: 0.5%Ni phosphor monitored at 1270 nm.

As a proof-of-concept experiment, we first synthesized a phosphor with persistent luminescence in the second near-infrared (NIR) window. In Fig. 1(a), non-doped ZnGa_2_O_4_ (ZGO) phosphor does not show any persistent phosphorescence after the irradiation, while Ni^2+^-doped ZnGa_2_O_4_ (ZGO: 0.5%Ni) phosphor exhibits a long persistent phosphorescence from 1050 to 1600 nm with peak at 1270 nm, covering a large part of the second NIR window. Also, a similar emission band is shown in the photoluminescence (PL) spectrum of ZGO: 0.5%Ni phosphor, which can be assigned to the ^3^T_2_(^3^F) → ^3^A_2_(^3^F) transition of octahedral Ni^2+^ ions^15^. ZnGa_2_O_4_ is a typical AB_2_O_4_-type spinel crystal. It’s more likely that Ni^2+^ ions are incorporated into the octahedral lattice sites, due to its similar ion radius with Ga^3+^ ion and the strong octahedral coordination preference^16^. Figure 1(b) shows the photoluminescence excitation (PLE) spectrum of ZGO: 0.5%Ni phosphor. The wavebands at 390 nm and 620 nm should be ascribed to the spin-allowed transitions of Ni^2+^, ^3^A_2_(^3^F) → ^3^T_1_(^3^P) and ^3^A_2_(^3^F) → ^3^T_1_(^3^F), respectively. Two small shoulders at about 440 nm and 690 nm correspond to the spin-forbidden transitions of Ni^2+^, ^3^A_2_(^3^F) → ^1^T_2_(^1^D) and ^3^A_2_(^3^F) → ^1^E(^1^D), respectively. These characteristic excitation peaks imply the octahedral coordination surrounding divalent nickel in ZGO: 0.5%Ni phosphor^17,18^. The attribution of ultraviolet excitation band is usually controversial. The strongest peak at 320 nm in ultraviolet region overlaps with Ni^2+^ [^3^A_2_(^3^F) → ^3^T_1_(^3^P)] band. To further effectively identify the ultraviolet excitation band, an afterglow excitation spectrum is measured. A distinct afterglow excitation band is shown in Fig. 1(b). It seemingly consists of two PLE peaks at 320 and 390 nm. Therefore, the ultraviolet excitation band at 320 nm should be assigned to the transition between ground state of Ni^2+^ to conduction band (CB), since capturing of an electron through the CB may be the most efficient path for the electron charging process^19,20^.

**Figure 2:**
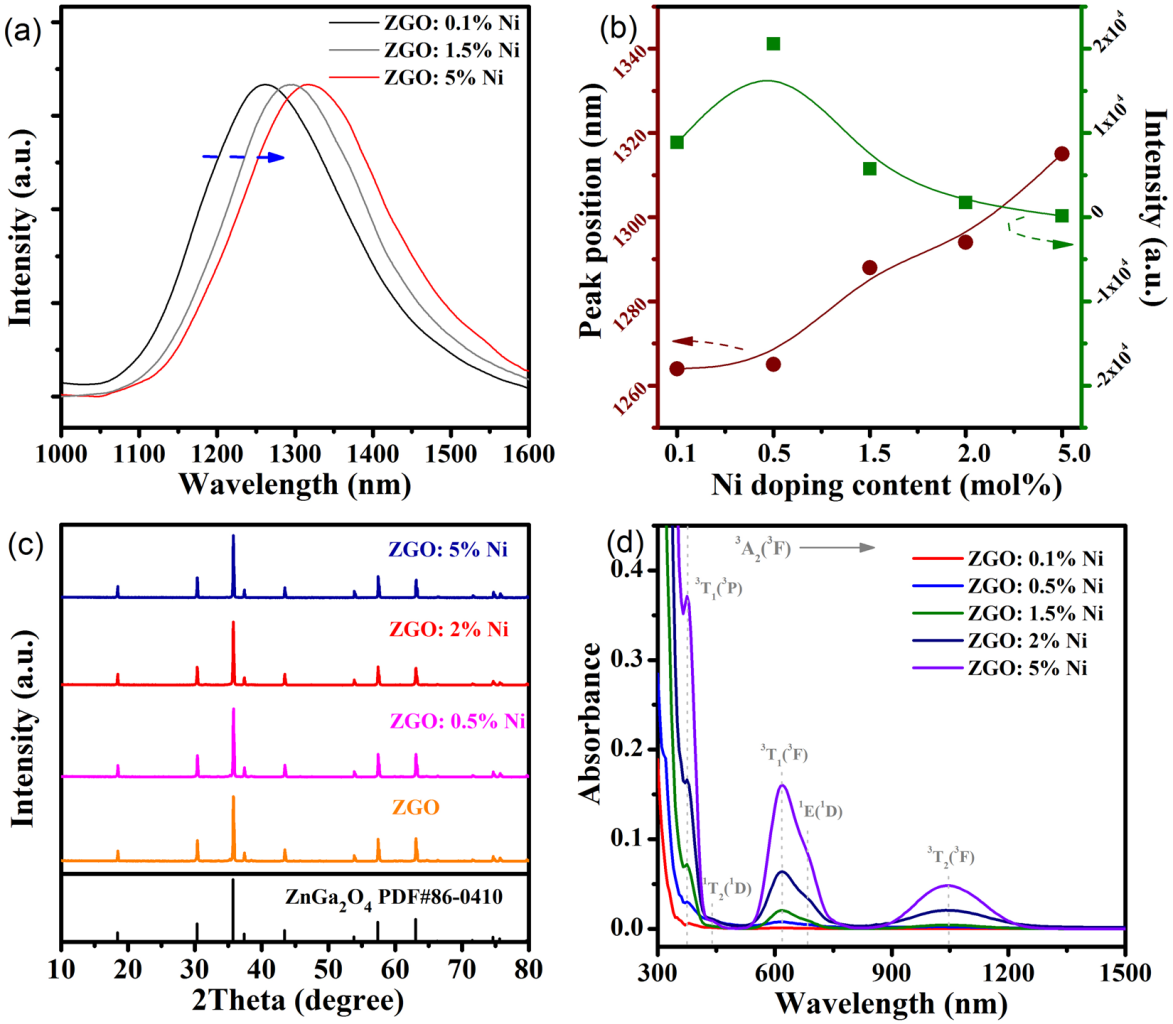
(**a**) Normalized afterglow spectra of ZGO: xNi^2+^ (x = 0.1%, 1.5%, 5%) phosphors after the irradiation by an ultraviolet lamp for 10 minutes. (**b**) Dependence of emission peak position (brown line) and afterglow intensity (green line) on Ni^2+^ doping concentrations. (**c**) X-ray diffraction patterns of ZGO: xNi^2+^ (x = 0%, 0.5%, 2%, 5%) phosphors. (**d**) Absorption spectra of ZGO: xNi^2+^ (x = 0.1%, 0.5%, 1.5%, 2%, 5%) phosphors.

Another interesting property of Ni^2+^-doped ZnGa_2_O_4_ phosphor is the spectra shift of PL (Supplementary Fig. S1) and afterglow band (Fig. 2(a)) as a function of Ni dopant concentration. With the increment of Ni^2+^ doping concentration, a red-shift of emission band is present, and the afterglow intensity first increases and then decreases, as shown in Fig. 2(b). Seemingly, 0.5% doping concentration of Ni^2+^ is suitable relatively. This variation of spectra shift according to the doping content of Ni^2+^ ions may be related to the energy transfer between neighboring Ni^2+^. The X-ray diffraction (XRD) of Ni^2+^-doped ZnGa_2_O_4_ phosphor is shown in Fig. 2(c), demonstrating that spectra shift has no concern with the crystal structure. To further validate if a possible reason for this phenomenon is because of energy difference between the electronic transitions of Ni^2+^, absorption spectra is often chosen as a tool^21^. In Fig. 2(d), the position of absorption peaks correspond to the transitions of Ni^2+^ in the octahedral sites^22^. Nevertheless, an overlap between the emission band at shorter wavelength region and the absorption band centered at 1045 nm may lead to the emission shift^23^. Because the distance between neighboring Ni^2+^ ions becomes shorter for higher Ni^2+^ concentration, the probability of energy transfer increases. The re-absorption by neighboring Ni^2+^ ions is suitable for elaborating the spectra shift in Fig. 2(a).

### Controlling long persistent phosphorescence via crystal field

**Figure 3:**
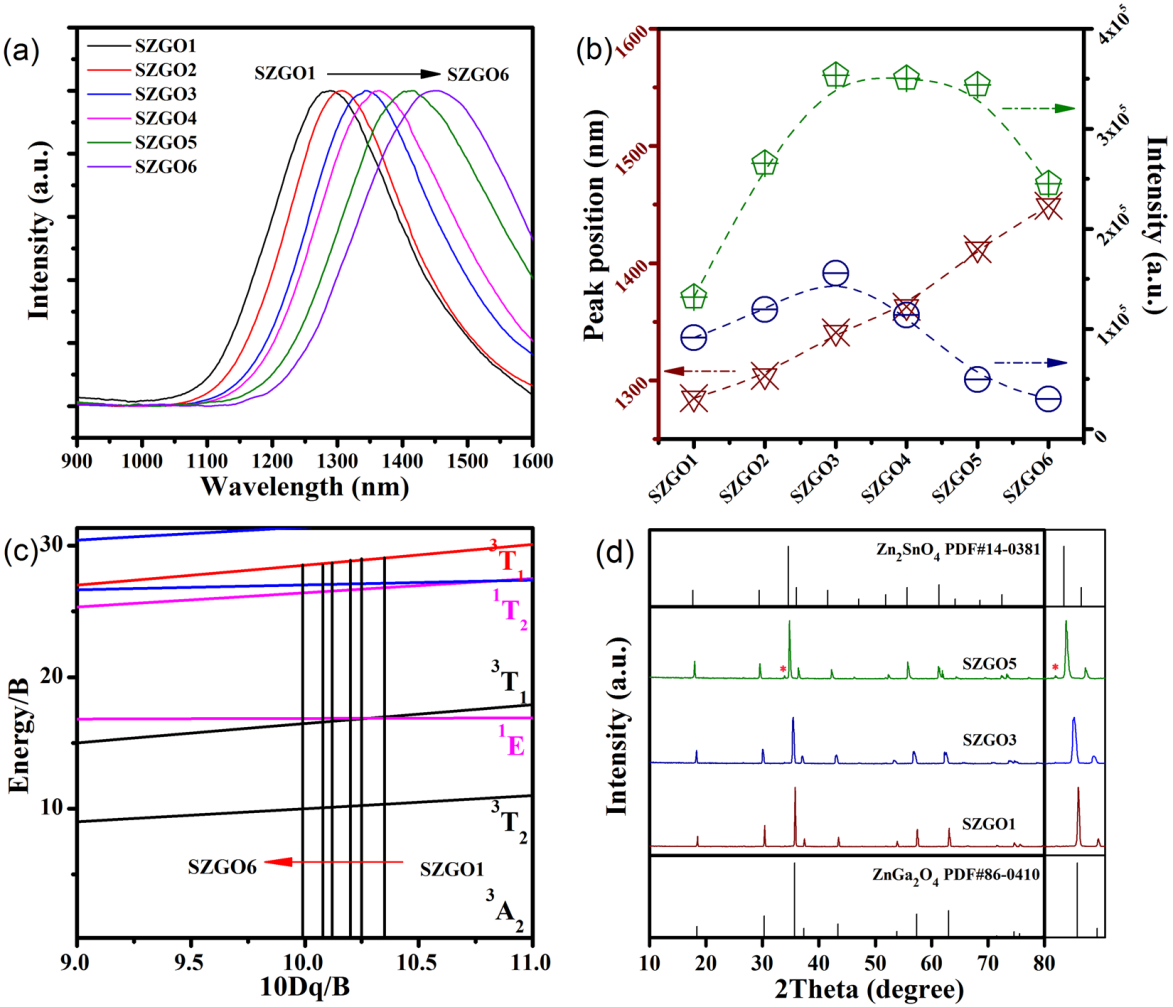
(**a**) Normalized afterglow spectra of the SZGO1-6 phosphors after the irradiation by an ultraviolet lamp for 10 minutes. (**b**) Dependence of emission peak positions (brown line), photoluminescence intensity (green line) and afterglow intensity (blue line) on the SZGO1-6 phosphors. (**c**) Tanabe–Sugano diagram in which the straight lines indicate the normalized crystal field of Ni^2+^ in the SZGO1-6 phosphors. (**d**) X-ray diffraction patterns of the SZGO1, SZGO3 and SZGO5 phosphors.

The hallmark of transition metal ions is that the electrons in their outmost d orbital strongly interact with their ligands and that the electronic configuration of the activation ions is affected strongly by the arrangement of surrounding ligands^13^. It is possible to control emission band via modulating the local crystal field surrounding Ni^2+^ ions. The radius of Sn^4+^ ion is similar as that of Ga^3+^ ion, and Sn^4+^ usually adapts to the octahedral configuration with the Sn-O distances (2.055 Å), close to Ga-O distances (1.98 Å), so Tin (Sn) might be a potential structural modifier to ZnGa_2_O_4_ host. As expected, the tunable PL and afterglow peak from 1270 to 1430 nm is observed in Zn_1+y_Sn_y_Ga_1.995−2y_O_4_:0.5% Ni^2+^ (where y = 0.05, 0.1, 0.3, 0.5, 0.7 and 0.9; designated as SZGO1, SZGO2, SZGO3, SZGO4, SZGO5 and SZGO6, respectively) phosphors (Supplementary Fig. S2 and Fig. 3(a)). Figure 3(b) displays the position variation of emission peak as a function of y value of Zn_1+y_Sn_y_Ga_1.995−2y_O_4_:0.5% Ni^2+^ phosphors: the higher y value achieves, the longer emission wavelength gets. In addition, both the PL and afterglow intensity increase firstly but drop later with increment of y value.

**Figure 4:**
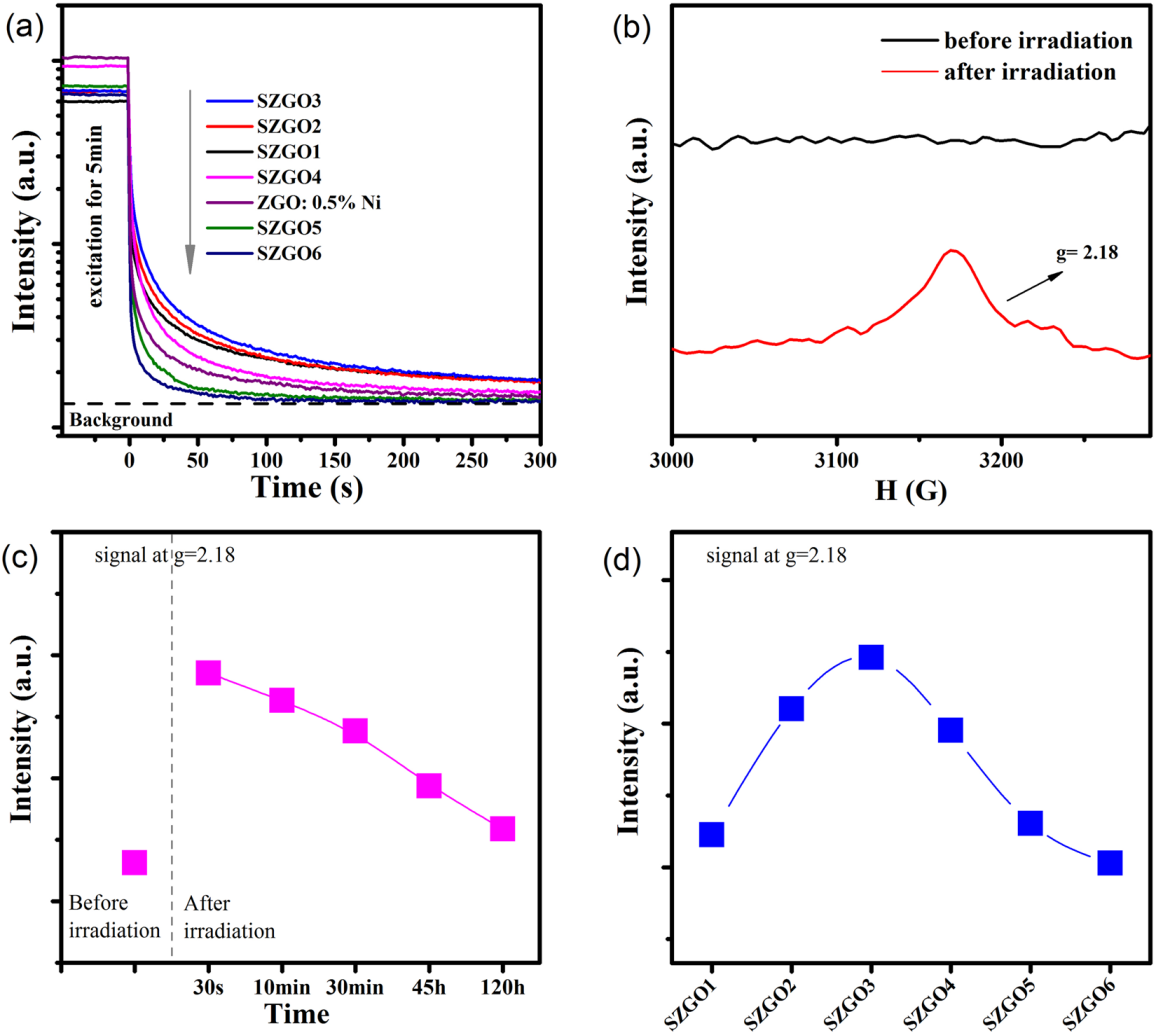
(**a**) Afterglow decay curves monitored at respective optimized emission wavelength of the SZGO1-6 phosphors and ZGO: 0.5% Ni (at 1270, 1305, 1345, 1365, 1410, 1430 and 1270 nm). All samples were pre-excited at 320 nm by a xenon lamp for 5 minutes. (**b**) EPR spectra of the SZGO2 phosphor before and after the irradiation by an ultraviolet lamp for 10 minutes. (**c**) Dependence of the intensity of the signal at g = 2.18 on time after ceasing the ultraviolet excitation for the SZGO2 phosphor. (**d**) Dependence of the intensity of the signal at g = 2.18 on the SZGO1-6 phosphors after ceasing the ultraviolet excitation.

In previous section, we acquire energy level distributions from absorption spectra (Supplementary Fig. S3), which can be applied for calculating the crystal field parameters of Ni^2+^ ions in these phosphors with the help of the Tanabe-Sugano matrix^24,25^. The crystal field splitting parameter (Dq) and the Racah parameter (B) can be estimated by spectroscopic data from the expression as follows:

1 $$10\,{\rm{Dq}}={{\rm{\nu }}}_{1}$$

2 $${\rm{B}}=({{\rm{\nu }}}_{2}^{2}+2{{\rm{\nu }}}_{2}^{1}-3{{\rm{\nu }}}_{1}{{\rm{\nu }}}_{2})/(15{{\rm{\nu }}}_{2}-27{{\rm{\nu }}}_{1})$$

where ν_1_ and ν_2_ are the wavenumbers of absorption bands corresponding to [^3^A_2_(^3^F) → ^3^T_2_(^3^F)] and [^3^A_2_(^3^F) → ^3^T_1_(^3^P)] transitions of Ni^2+^ ions, respectively. All obtained crystal field parameters are given in Table 1 and Fig. 3(c). Based on the experimental data, the crystal field (10 Dq/B) is weakened gradually with the higher concentration of Sn and Zn. Lower crystal field contributes to a decreasing in the transition energy from the excited state levels to the ground state level, which is in accordance with the red shift of experimental variation both in spectroscopic data. Zhou *et al*.^26^ have investigated the infrared-luminescent Ni^2+^-activated materials with tunable and ultra-broadband luminescent characteristics. In their research, they demonstrated that both the distribution of crystal anions and the size of coordination polyhedrons play an important role in tuning the optical properties of central ion. According to the X-ray diffraction patterns in Fig. 3(d), the diffraction peaks shift to lower angles at higher y value, indicating the lattice expanded gradually because of the formation of solid solution in terms of Bragg’s law. An excess of SnO_2_ leads to the appearance of an unidentified peak with an asterisk in high Sn substituted sample. The crystal phases of Ni^2+^-doped Zn_1+y_Sn_y_Ga_1.995−2y_O_4_ phosphors vary from normal spinel to inverse spinel with the increasing of y value^27^. In Zn_2_SnO_4_, an inverse spinel crystal, Zn^2+^ ions are distributed in the octahedral and the tetrahedral sites whereas Sn^4+^ ions occupy octahedral sites. The smaller Ga^3+^ (62 pm) ions are substituted by both Sn^4+^ (69 pm) and Zn^2+^ (74 pm) ions, so the Ni^2+^-O^2−^ distance is lengthened and the degree of splitting energy decreases^28^. As a result, a decrease of energy splitting and a red spectral shift is dependent on the Sn and Zn doping content. Figure 4(a) shows the afterglow decay curves of the SZGO1-6 and ZGO: 0.5%Ni phosphors. The afterglow time increases at the beginning, and then decreases. In general, long persistent phosphorescence is closely associated with the migration of dynamic carrier among trapping states. Electron paramagnetic resonance (EPR) can identify the variation, especially for the unpaired electron^14,16,29^. The value of g is the primary empirical parameter that characterizes the response of a paramagnetic molecule. A new signal at about g = 2.18 appears after the ultraviolet irradiation in the EPR spectra of all Ni^2+^-doped samples, implying the existence of a number of detectable unpaired electrons (Fig. 4(b) and Supplementary Fig. S6). Meanwhile, this new signal at g = 2.18 attenuates after stopping of irradiation, quickly at first, and then slowly with time, which is consistent with the afterglow degradation (Fig. 4(c)). These results may suggest that Ni^2+^ participates in the persistent duration as the trap center^30,31^. The intensity variation of the signal at g = 2.18 of different samples after ceasing the ultraviolet excitation is shown in Fig. 4(d). The result demonstrates that SZGO3 phosphor has a suitable trap structure. As discussed above, with the increasing of Zn and Sn content substitution for Ga in Zn_1+y_Sn_y_Ga_1.995−2y_O_4_: 0.5% Ni^2+^, the crystal field around Ni^2+^ becomes smaller. A larger Ni^2+^-O^2−^ distance along with the lattice expand leads to the tunable persistent phosphorescence in the second NIR window. The persistent duration determined by trapping state has also been affected.

**Table 1:** Estimated crystal field parameters of Ni^2+^ in the Zn_1+y_Sn_y_Ga_1.995−2y_ O_4_: 0.5% Ni^2+^ (where y = 0.05, 0.1, 0.3, 0.5, 0.7 and 0.9; designated as SZGO1, SZGO2, SZGO3, SZGO4, SZGO5 and SZGO6, respectively) phosphors.

Sample	ν_1_ [cm^−1^]	ν_2_ [cm^−1^]	Dq [cm^−1^]	B [cm^−1^]	10 Dq/B
SZGO1	9652	27100	965	932	10.35
SZGO2	9560	26954	956	932	10.25
SZGO3	9461	26738	946	927	10.20
SZGO4	9302	26385	930	919	10.12
SZGO5	9116	25907	911	905	10.08
SZGO6	8897	25381	884	890	9.99

ν_1_ and ν_2_ are the wavenumbers of absorption bands corresponding to [^3^A_2_(^3^F) → ^3^T_2_(^3^F)] and [^3^A_2_(^3^F) → ^3^T_1_(^3^P)] transitions of Ni^2+^ ions, respectively. Dq and B mean the crystal field splitting and the Racah parameter^25^.

### Near-infrared imaging in pork tissues

For a proof-of-concept demonstration of bioimaging, a preliminary NIR-imaging in pork tissue of the SZGO3 phosphor is provided, using a supersensitive camera in dark room (Supplementary Fig. S9). Figure S9(b–e) shows the images taken at the same location at different time intervals. The signal detected from the phosphor could clearly distinguish from autofluorescence of the tissue in real-time observation. The phosphor gives a strong signal at first and then decreases with time. The afterglow signal is detectable for 10 minutes with high resolution in the sensitivity limit of the system measurement. It is shown that this NIR phosphorescent material has the possibility of potentially being applied in advanced bioimaging. However, further research is still underway on nanocrystallization and functionalization of these phosphors.

## Conclusions

In summary, we demonstrate ZnGa_2−x_O_4_: xNi^2+^ and Zn_1+y_Sn_y_Ga_2−x−2y_O_4_: xNi^2+^ long persistent phosphors (LPPs), which feature an emission band from 1050 to 1600 nm in the second near-infrared (NIR) window. By means of increasing Zn and Sn substitution content for Ga, the exceptional peak shift of ultrabroadband NIR persistent phosphorescence can be tuned from 1270 to 1430 nm via controlling the crystal field around Ni^2+^. Furthermore, the characteristic operational waveband and excellent tunability offer the possibility of these NIR phosphorescent materials as multifunctional optical materials applying for visualizing structural and functional process in cells, tissues and other complex system.

## Methods

### Materials

4 N Pure ZnO, Ga_2_O_3_, SnO_2_ and NiO were selected as raw materials.

### Synthesis

Phosphors with molar composition of ZnGa_2−x_O_4_: xNi^2+^ (x = 0, 0.1%, 0.5%, 1%, 1.5%, 2%, 5%) and Zn_1+y_Sn_y_Ga_1.995−2y_O_4_: 0.5% Ni^2+^ (y = 0.05, 0.1, 0.3, 0.5, 0.7, 0.9), (Supplementary Table S1) were prepared by conventional high temperature solid state reaction method. The stoichiometric stating materials were thoroughly homogenized by using an agate pestle and mortar for grinding, the mixture was transferred into an alumina crucible and then sintered at 1873 K for 3 hours in air included two heating processes with different heating rates (that is, below 1073 K at 5 K/minutes, from 1073 K to 1873 K at about 3 K/minutes). After that, the samples were furnace-cooled to room temperature and ground again into powder for subsequent use.

### Measurements and characterization

The prepared materials were analyzed by X-ray diffraction (XRD) (Cu/Kα) and the data was collected between 10° and 80°. The morphology and particle size of the prepared phosphors were observed by Nova Nano SEM 430 Scanning Electron Microscopy (SEM, Supplementary Fig. S10). Room-temperature photoluminescence (PL) spectra, photoluminescence excitation (PLE) spectra, afterglow spectra and decay curves were measured with a high resolution spectrofluorometer (UK, Edinburgh Instruments, FLS920) equipped with a 500 W xenon lamp as an excitation source, with a Hamamatsu R928P visible photomultiplier (250–850 nm) and a liquid nitrogen-cooled Hamamatsu R5509-72 NIR photomultiplier as the detectors. The afterglow excitation spectra were extracted from afterglow decay curves monitored at 1270 nm after different excitation wavelengths. The phosphor was pre-irradiated for 5 minutes by xenon lamp between 250 nm to 750 nm in 10 nm or 20 nm steps. Subsequently, we took afterglow intensity I_10s_ as a function of the excitation wavelengths over the spectral range of 250–750 nm, I_10s_ means the afterglow intensity recorded at ten seconds after irradiation ended. The absorption spectra were measured by PerkinElmer Lambda 950 spectrometer in the region of 300–1500 nm. Electron paramagnetic resonance (EPR) spectra were recorded by using a Bruker E500 EPR spectrometer operating at X-band microwave frequency for studying paramagnetic defects. The samples were pre-annealed at 773 K for 10 minutes before testing, which were measured both before and after the irradiation by an ultraviolet lamp for 10 minutes.

### Imaging

Near-infrared (NIR) persistent luminescent tissue imaging were performed with a modified imaging system, using a Germany Goldeye P-008 NIR camera as the signal collector. The phosphor was spread on the pork tissue at the depth of 3 mm. The used pork with a size of 14 mm × 20 mm × 5 mm was bought from the supermarket. All images were taken in a dark room under the camera exposure time of 1000 milliseconds after excitation by an ultraviolet lamp for 10 minutes, and analyzed with home-made software.

## Electronic supplementary material

supplementary information
