# Publisher Correction: Tunable long persistent luminescence in the second near-infrared window via crystal field control

**DOI:** 10.1038/s41598-020-61344-0

**Published:** 2020-03-06

**Authors:** Jianmin Nie, Yang Li, Shanshan Liu, Qiuqun Chen, Qi Xu, Jianrong Qiu

**Affiliations:** 1 State Key Laboratory of Luminescent Materials and Devices, School of Materials Science and Technology, South China University of Technology, Guangzhou, 510640 China; 2 Guangdong Provincial Key Laboratory of Fiber Laser Materials and Applied Techniques, South China University of Technology, Guangzhou, 510640 China; 3 School of Physics and Optoelectronic Engineering, Guangdong University of Technology, Guangzhou, 510006 China; 4 State Key Laboratory of Modern Optical Instrumentation, College of Optical Science and Engineering, Zhejiang University, Hangzhou, Zhejiang, 310027 China

Correction to: *Scientific Reports* 10.1038/s41598-017-12591-1, published online 29 September 2017

This Article contains an error in Equation 2.

$$B=({v}_{2}^{2}+2{v}_{2}^{1}-3{v}_{1}{v}_{2})/(15{v}_{2}-27{v}_{1})$$

should read:

$$B=({v}_{2}^{2}+2{v}_{1}^{2}-3{v}_{1}{v}_{2})/(15{v}_{2}-27{v}_{1})$$

