# Supplementary material for: Tunable long persistent luminescence in the second near-infrared window via crystal field control
Source: Sci Rep. 2017 Sep 29;7:12392. doi: 10.1038/s41598-017-12591-1 (PMC5622036; doi:10.1038/s41598-017-12591-1)
Supplement: Supplementary file 1 — supplementary information [file 41598_2017_12591_MOESM1_ESM.pdf]

Tunable long persistent luminescence in the second near-infrared window via crystal field control

Jianmin Nie[1,2], Yang Li[1,3,*], Shanshan Liu[1,2], Qiuqun Chen[1,2], Qi Xu[1,2] and Jianrong Qiu[1,2,4,*]

[1] State Key Laboratory of Luminescent Materials and Devices , School of Materials Science and Technology, South China University of Technology, Guangzhou 510640, China

[2] Guangdong Provincial Key Laboratory of Fiber Laser Materials and Applied Techniques, South China University of Technology, Guangzhou 510640, China

[3] School of Physics and Optoelectronic Engineering, Guangdong University of Technology, Guangzhou 510006, China

[4] State Key Laboratory of Modern Optical Instrumentation, College of Optical Science and Engineering, Zhejiang University, Hangzhou, Zhejiang 310027, China

*<u>**Corresponding authors:**</u> qjr@scut.edu.cn; lychris@sina.com

Fax: +86-20-87114204; Tel: +86-20-87113646;

Supplementary Information

Figures Legends

Figure S1. Normalized photoluminescence spectra of $ZnGa_{2-x}O_4$: xNi^{2+} (x=0.1%, 1.5%, 5%) phosphors.

Figure S2. Normalized photoluminescence spectra of the SZGO1-6 phosphors.

Figure S3. Absorption spectra of the SZGO1-6 phosphors, the inset are the normalized absorption spectra ranged from 850 nm to 1400 nm.

Figure S4. Afterglow decay curves in 10 minutes scale monitored at respective optimized emission wavelength (that is, 1270, 1305, 1345, 1365, 1410, and 1430 nm, respectively) of the SZGO1-6 phosphors. All samples were pre-excited at 320 nm by a xenon lamp for 5 minutes.

Figure S5. EPR spectra of the $ZnGa_{2-x}O_4$: xNi^{2+} (x=0.1%, 0.5%, 1%) phosphors after the irradiation by an ultraviolet lamp for 10 minutes at room temperature.

Figure S6. EPR spectra of $Zn_{1+y}Sn_yGa_{1.995-2y}O_4$: 0.5%$Ni^{2+}$ (y=0.05, 0.1, 0.3, 0.5, 0.7, 0.9; corresponding to SZGO1-SZGO6, respectively) phosphors before and after irradiation by an ultraviolet lamp for 10 minutes at room temperature.

Figure S7. (a) Dependence of the intensity of the signal at g=1.97 on time after ceasing the ultraviolet excitation for the SZGO2 phosphor. (b) Dependence of the intensity of the signal at g=4.31 on time after ceasing the ultraviolet excitation for the SZGO3 phosphor. (c) Dependence of the intensity of the signal at g=11.57 on time after ceasing the ultraviolet excitation for the SZGO2 phosphor.

Figure S8. EPR spectra of the SZGO2 phosphor at different time intervals (30 s, 10 min, 30 min, 45 h, and 120 h) after ceasing the ultraviolet excitation are measured. (a) EPR signal at g=2.18; (b) EPR signal at g=11.57.

Figure S9. Application in tissue imaging. (a) Digital photos of pork tissue for tissue imaging. (b-e) NIR imaging of pork tissue for an injection of the SZGO3 phosphors at the depth of 3 mm at different time intervals (5 s, 1 min, 5 min, 10 min). The sample was pre-excited by an ultraviolet lamp for 10 minutes.

Figure S10. SEM image of $ZnGa_{1.995}O_4$: 0.5% Ni^{2+} phosphor.

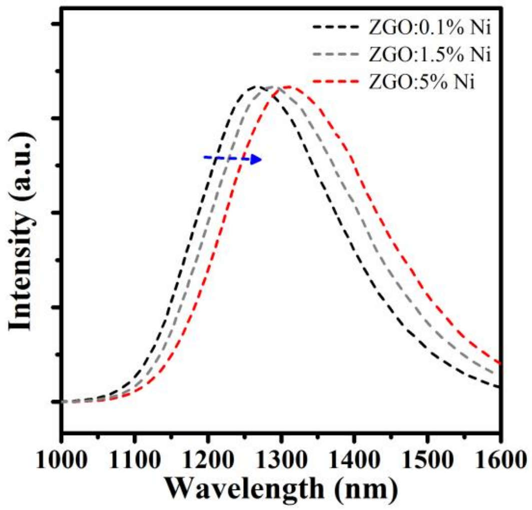
ZGO:0.1% Ni
ZGO:1.5% Ni
ZGO:5% Ni
Intensity (a.u.)
1000
1100
1200
1300
1400
1500
1600
Wavelength (nm)

Figure S1

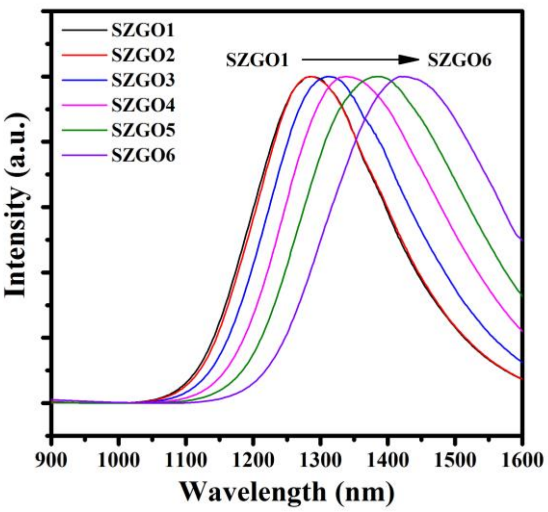
SZGO1
SZGO2
SZGO3
SZGO4
SZGO5
SZGO6
SZGO1
SZGO6
Intensity (a.u.)
900
1000
1100
1200
1300
1400
1500
1600
Wavelength (nm)

Figure S2

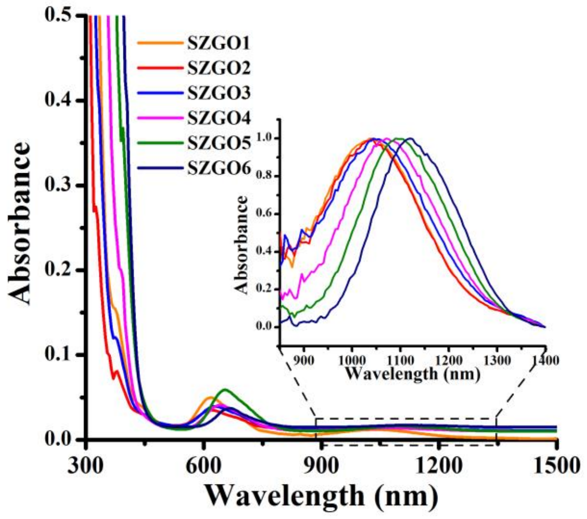
SZGO1
SZGO2
SZGO3
SZGO4
SZGO5
SZGO6
Absorbance
Wavelength (nm)
Absorbance
Wavelength (nm)

Figure S3

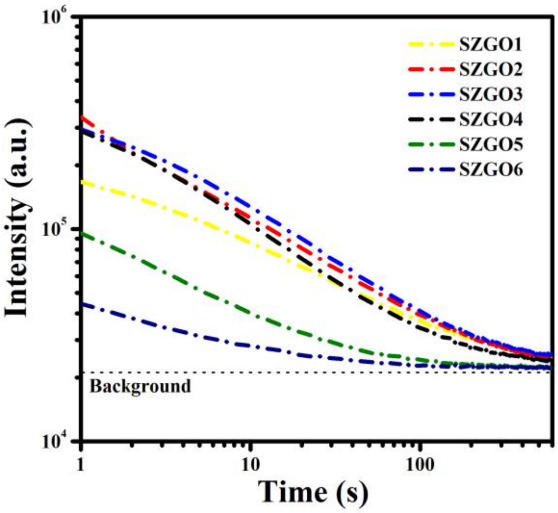

SZGO1
SZGO2
SZGO3
SZGO4
SZGO5
SZGO6
Intensity (a.u.)
Background
Time (s)

Figure S4

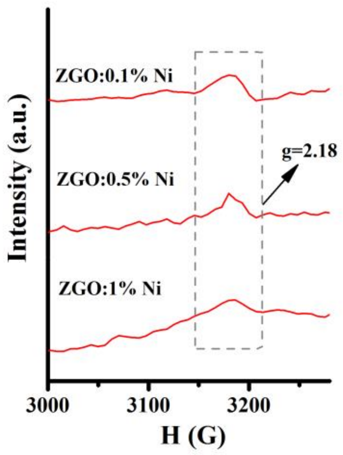

Figure S5

A new signal at g=2.18 appears after the ultraviolet irradiation in the EPR spectra of samples of different Ni^{2+}-doped concentrations, implying the existence of a number of detectable unpaired electrons. The intensity of the signal at g=2.18 weakens at high doping concentration. Its variation is consistent with that of afterglow intensity. It may further indicate that Ni^{2+} participates in the persistent duration as the trap center.

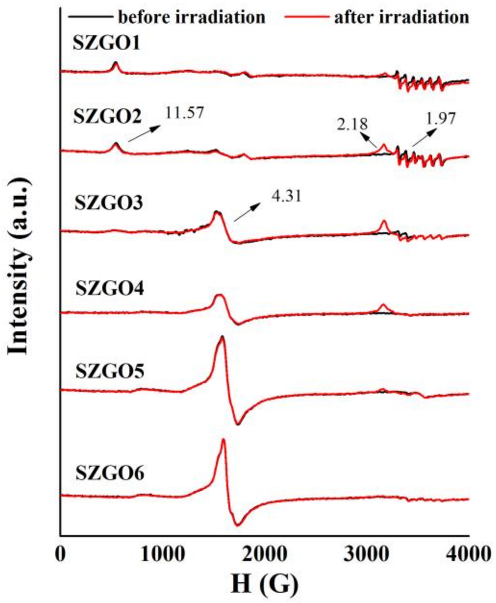

Figure S6

As shown in Fig. S6, a new signal at about g=2.18 appears after the ultraviolet irradiation in the EPR spectra of all samples. The signal at g=1.97 and below is characteristic of Mn^{2+} as impurity. Mn^{2+} at the level of ppm easily exists in the raw material of transition ion, and they also can be easily detected by EPR.

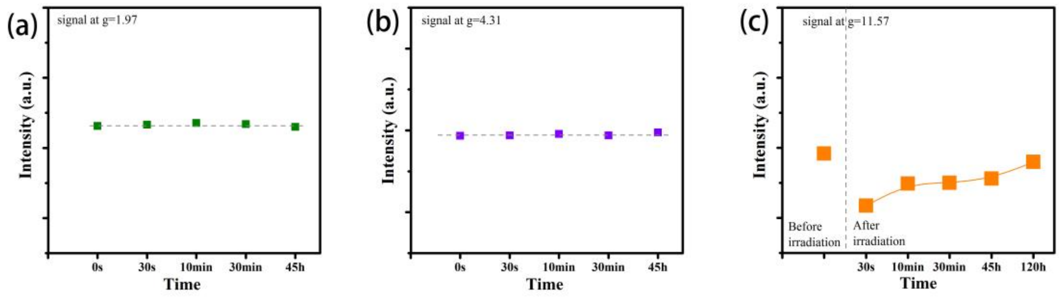

Figure S7

According to the time-dependent EPR spectra of Mn^{2+} signal (Fig. S7(a)), we find that Mn^{2+} does not participate the decay duration. Meanwhile, according to the time-dependent EPR spectra at g=4.31 as shown in Fig. S7(b), we also find that the signal intensity keeps unchanged, implying that the consideration of afterglow process do not involve this EPR signal. Interestingly, we find that the signal at g=11.57 displays an opposite trend in comparison with variation of the signal at g=2.18 (Fig. S7(c)), which decreases substantially due to irradiation but increases with time after stopping of irradiation. These results may give insight into the distinct electron interchange reaction between two delocalized traps during afterglow degradation.

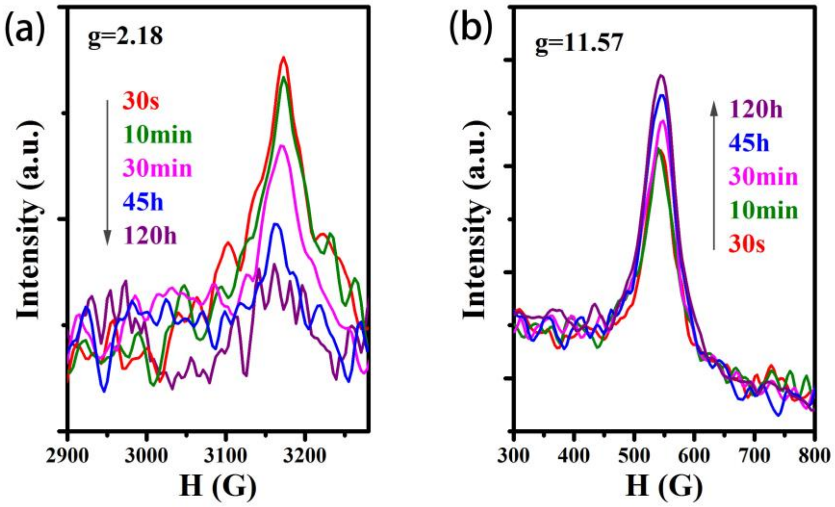
(a)
g=2.18
30s
10min
30min
45h
120h
Intensity (a.u.)
2900
3000
3100
3200
H (G)
(b)
g=11.57
120h
45h
30min
10min
30s
Intensity (a.u.)
300
400
500
600
700
800
H (G)

Figure S8

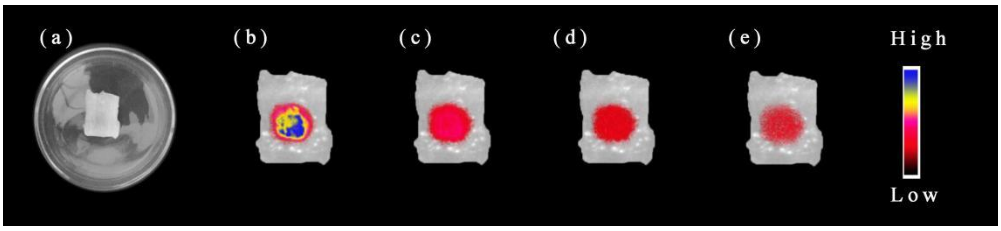

Figure S9

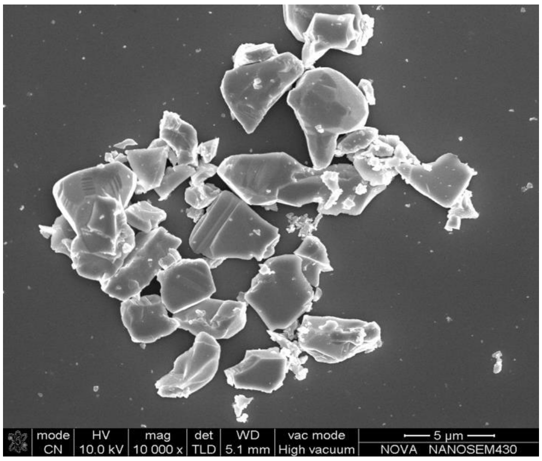
mode
CN
HV
10.0 kV
mag
10 000 x
det
TLD
WD
5.1 mm
vac mode
High vacuum
5 μm
NOVA NANOSEM430

Figure S10

Tables

Table S1 Chemical compositions of $ZnGa_{2-x}O_4$: xNi^{2+} (x=0, 0.1%, 0.5%, 1%, 1.5%, 2%, 5%) and $Zn_{1+y}Sn_yGa_{1.995-2y}O_4$: $0.5\%Ni^{2+}$ (y=0.05, 0.1, 0.3, 0.5, 0.7, 0.9) phosphors.

Table S1 Chemical compositions of $ZnGa_{2-x}O_4$: xNi^{2+} (x=0, 0.1%, 0.5%, 1%, 1.5%, 2%, 5%) and $Zn_{1+y}Sn_yGa_{1.995-2y}O_4$: 0.5%$Ni^{2+}$ (y=0.05, 0.1, 0.3, 0.5, 0.7, 0.9) phosphors.

No	Composition	No	Composition
ZGO	$ZnGa_2O_4$	SZGO1	$Zn_{1.05}Sn_{0.05}Ga_{1.895}O_4$: 0.5%$Ni^{2+}$
ZGO: 0.1%Ni	$ZnGa_{1.999}O_4$: 0.1%Ni^{2+}	SZGO2	$Zn_{1.1}Sn_{0.1}Ga_{1.795}O_4$: 0.5%$Ni^{2+}$
ZGO: 0.5%Ni	$ZnGa_{1.995}O_4$: 0.5%Ni^{2+}	SZGO3	$Zn_{1.3}Sn_{0.3}Ga_{1.395}$ O_4: 0.5%Ni^{2+}
ZGO: 1%Ni	$ZnGa_{1.99}O_4$: 1%Ni^{2+}	SZGO4	$Zn_{1.5}Sn_{0.5}Ga_{0.995}$ O_4: 0.5%Ni^{2+}
ZGO: 1.5%Ni	$ZnGa_{1.985}O_4$: 1.5%Ni^{2+}	SZGO5	$Zn_{1.7}Sn_{0.7}Ga_{0.595}$ O_4: 0.5%Ni^{2+}
ZGO: 2%Ni	$ZnGa_{1.98}O_4$: 2%Ni^{2+}	SZGO6	$Zn_{1.9}Sn_{0.9}Ga_{0.195}$ O_4: 0.5%Ni^{2+}
ZGO: 5%Ni	$ZnGa_{1.95}O_4$: 5%Ni^{2+}		
